# 1660. Dalbavancin Utilization Registry Investigating Value and Effectiveness (DRIVE): Outcomes Report on Real-World Use

**DOI:** 10.1093/ofid/ofac492.126

**Published:** 2022-12-15

**Authors:** Bruce M Jones, Kerry O Cleveland, Pedro L Gonzalez, Urania Rappo, Todd Riccobene, Rosie D Lyles

**Affiliations:** St. Joseph's/Candler Health System, Savannah, Georgia; University of Tennessee Health Science Center, Memphis, Tennessee; Becton Dickinson, Franklin Lakes, New Jersey; BiomX, Branford, Connecticut; AbbVie Inc, Madison, New Jersey; AbbVie, Evanston, Illinois

## Abstract

**Background:**

Dalbavancin (dalba), a long-acting lipoglycopeptide approved by the US FDA and EMA for acute bacterial skin and skin structure infections (ABSSSI) has potent activity against Gram-positive pathogens including MRSA. We describe the real-world use of dalba in patients with ABSSSI, bloodstream infections (BSI), bone and joint infection (BJI), or other infections (OI) from a retrospective registry study of dalba.

**Methods:**

Dalbavancin Utilization Registry Investigating Value and Effectiveness (DRIVE) was a phase 4 observational, multicenter, retrospective cohort study of the real-world use of dalba in adults geographically spread across the US. Patients were treated entirely at the physicians’ discretion. Data were collected from 25 Mar 2017–27 Nov 2018 and included patient demographics, diagnosis, microbiology, antibiotic use, clinical outcome, and safety until 60 days after last dalba dose. A post hoc analysis applied definitions for clinical success and failure similar to the dalba phase 3 clinical trials.

**Results:**

The Safety Population comprised 1168 patients treated at 31 healthcare sites. Of 957 evaluable patients, 815 (85.2%) had ABSSSI, 48 (5.0%) had BSI, 87 (9.0%) had BJI, and 7 (0.7%) had OI. Clinical success was achieved in 91.7%, 89.6%, 86.2%, and 100% of patients with ABSSSI, BSI, BJI, and OI, respectively (Figure 1). Median cumulative dose of dalba for the index infection was 1500 mg for all categories, most commonly as 1 infusion (86.7% ABSSSI, 63.1% BSI, 41.4% BJI, 37.5% OI; Figure 2). 36.9% of patients with BSI and 58.6% with BJI received ≥ 2 infusions. Most patients were treated as outpatients (87.8%, 76.9%, 89.2%, 100%, with ABSSSI, BSI, BJI, and OI, respectively). Mean (SD) total cost of care associated with dalba use (medications, hospital stay, surgeries/procedures) was 5261.2 (1604.55), 7710.5 (7144.1), 8393.5 (5296.8), and 6305.8 (3338.1) USD for patients with ABSSSI, BSI, BJI, and OI, respectively. Adverse events evaluated as possibly related to dalba were reported in 33/1168 (2.8%) patients: 29 (2.9%) with ABSSSI, and 4 (3.6%) with BJI.

**Conclusion:**

Dalba demonstrated high rates of clinical success and safety in the real-world setting when used to treat patients for suspected or confirmed Gram-positive infections.

**Disclosures:**

**Bruce M. Jones, Pharm.D., FIDSA, BCPS**, AbbVie: Advisor/Consultant|AbbVie: Honoraria|La Jolla: Honoraria|Melinta: Advisor/Consultant|Paratek: Honoraria|Regeneron: Honoraria **Kerry O. Cleveland, MD**, AbbVie: Honoraria|Cumberland: Honoraria|Merck: Honoraria|Pfizer: Honoraria **Pedro L. Gonzalez, MD, MT**, AbbVie: Employee of AbbVie at the time of study conduct and analysis.|AbbVie: Stocks/Bonds|Becton Dickinson: Employee **Urania Rappo, MD**, AbbVie: Employee of Allergan, before its acquisition by AbbVie, at the time of study conduct and analysis|BiomX: Employee **Todd Riccobene, PhD**, AbbVie: Employee|AbbVie: Stocks/Bonds **Rosie D. Lyles, MD, MHA, MSc**, AbbVie: AbbVie Employee|AbbVie: Stocks/Bonds.

